# Compensating Level-Dependent Frequency Representation in Auditory Cortex by Synaptic Integration of Corticocortical Input

**DOI:** 10.1371/journal.pone.0169461

**Published:** 2017-01-03

**Authors:** Max F. K. Happel, Frank W. Ohl

**Affiliations:** 1 Leibniz Institute for Neurobiology, D-39118, Magdeburg, Germany; 2 Institute of Biology, Otto-von-Guericke-University, D-39120 Magdeburg, Germany; 3 Center for Behavioral Brain Sciences (CBBS), Magdeburg, Germany; Universidad de Salamanca, SPAIN

## Abstract

Robust perception of auditory objects over a large range of sound intensities is a fundamental feature of the auditory system. However, firing characteristics of single neurons across the entire auditory system, like the frequency tuning, can change significantly with stimulus intensity. Physiological correlates of level-constancy of auditory representations hence should be manifested on the level of larger neuronal assemblies or population patterns. In this study we have investigated how information of frequency and sound level is integrated on the circuit-level in the primary auditory cortex (AI) of the Mongolian gerbil. We used a combination of pharmacological silencing of corticocortically relayed activity and laminar current source density (CSD) analysis. Our data demonstrate that with increasing stimulus intensities progressively lower frequencies lead to the maximal impulse response within cortical input layers at a given cortical site inherited from thalamocortical synaptic inputs. We further identified a temporally precise intercolumnar synaptic convergence of early thalamocortical and horizontal corticocortical inputs. Later tone-evoked activity in upper layers showed a preservation of broad tonotopic tuning across sound levels without shifts towards lower frequencies. Synaptic integration within corticocortical circuits may hence contribute to a level-robust representation of auditory information on a neuronal population level in the auditory cortex.

## Introduction

Sensory systems have evolved to support robust perception of sensory objects. In auditory perception, this robustness is evident in the perceptual constancy of auditory objects despite sound inputs not belonging to the object in question (e.g. competing objects or noise) or the large variance of sound parameters (e.g. sound source localization or sound level).

Studies at the level of single neurons have shown that the activity of the vast majority of neurons across the entire peripheral and central auditory system dependent on sound level [1–4]. However, level-tolerant single unit activity has also been reported [5,6] and the interaction of the effects of signal-to-noise ratio and overall sound level was recently described [7]. Most studies on level-constancy of auditory representations are based on single-unit data. However, physiological correlates should be manifest on the level of larger neuronal assemblies or population activity. There is evidence that adaptive gain modulation of neurons or neuronal populations contribute to the mechanisms underlying perceptual robustness [8–11]. This is further indicated by approaches reconstructing population activity patterns [9,12] or population-based information [13] from single-unit data or by approaches identifying discernible information contributions of single-unit and population activity in complex sound representation [14,15]. In this respect, frequency representations in the auditory cortex can span up to several octaves on the level of individual neurons [16–18] and of population activity [19] in dependence of the sound level. However, the underlying functional circuit mechanisms of sound parameter representation in primary auditory cortex are still elusive.

In this study, we therefore used current-source density recordings (CSD) to investigate sound level-dependent processing in primary auditory cortex field AI of anaesthetized Mongolian gerbils (*Meriones unguiculatus*). With increasing sound level, strongest leading early synaptic inputs were evoked consistently with lower frequencies, as compared to the characteristic frequency (CF) at response threshold. By employing pharmacological silencing of corticocortically relayed activity [20,21] we found that this level-dependent shift of synaptic population tuning at stimulus onset was mainly inherited from thalamocortical inputs. A recently established method allows to dissociate thalamocortically relayed from corticocortically relayed input to a cortical patch based on the analysis of the relative residuum of the CSD [20]. Thereby, we could demonstrate that, above threshold, lateral inputs at a given cortical patch provide early spectral information about the CF independent of the sound level. Various studies investigating spiking characteristics have found similar level-dependent shifts of the response tuning on the level of individual neurons across the peripheral [3], subcortical [4], and cortical [6,18,22] auditory system. These findings challenge the use of frequency tuning at response threshold in order to define the tonotopic region under investigation. We will discuss how current findings across the auditory system relate to intensity tolerant coding of sound frequency. Further, tone-evoked corticocortical synaptic activity in supragranular layers did not display corresponding level-dependent tuning shifts. We therefore suggest that corticocortical synaptic integration supports a stable tonotopic representation across ranges of stimulus intensity mainly beyond layers of direct thalamocortical input.

## Materials and Methods

Experiments were performed on 8 adult male ketamine-xylazine anesthetized Mongolian gerbils (*Meriones unguiculatus*) (age: 3–16 months, body weight: 80–120 g). Surgical and experimental procedures have been described in detail previously [20]. All experiments were conducted in accordance with the international NIH Guidelines for Animals in Research and with ethical standards for the care and use of animals in research defined by the German Law for the protection of experimental animals. Experiments were approved by an ethics committee of the state Saxony-Anhalt, Germany.

### Surgery and recordings

Mongolian gerbils were anesthetized by intraperitoneal infusion (0.06ml/h) of 45% ketamine (50 mg/ml, Ratiopharm, Germany), 5% xylazine (Rompun, 2%, BayerVital, Germany) and 50% isotonic sodium chloride solution (154mmol/l, Braun, Germany). Status of anesthesia was monitored and body temperature was kept at 37°C. The right auditory cortex was exposed by craniotomy (~3x4mm) of the temporal bone. Recordings were performed in an acoustically and electrically shielded recording chamber. Laminar profiles of local field potentials (LFP) were measured using custom-made linear multi-channel shaft electrodes (24–28 channels; 55–75 μm spacing) inserted perpendicular to the cortical surface (for further details see Happel et al., 2010). Recorded potentials were preamplified (500x), band-pass filtered between 3–170 Hz (3 dB cut-off frequency), digitized at 2 kHz (Multichannel Acquisition Processor, Plexon Inc.) and averaged over 100 stimulus repetitions. The field AI in primary auditory cortex was identified by vasculature landmarks and physiological parameters [23,24]. Pseudo-randomized series of pure tones (200 ms with 5 ms sinusoidal rising and falling ramps; spanning 8 octaves from 125 Hz to 16 kHz; inter-stimulus interval: 800 ms) were digitally synthesized using Matlab (Natwick, MA). These were converted to analog signals by a data acquisition card (NI PCI-BNC2110; National Instruments, Germany). Stimuli were delivered via a programmable attenuator (g.PAH, Guger Technologies; Austria), an amplifier (STAX SRM-3) and an electrostatic headphone (STAX SR lambda professional) positioned 3 cm in front of the animal’s head. Sound intensities varied between 10 and 80 dB SPL.

### Pharmacological silencing of corticocortically relayed activity

Corticocortically relayed activity was blocked by topical application of 20 μl onto the surface of the cortex of the GABA_A_-agonist muscimol hydrobromide (Sigma). Concentration was varied between of 0.2–1.0 μg/μl (max. 8.4 mM) to effectively block activity of all cortical layers (Happel et al., 2010) without affecting subcortical structures [25]. Cortical silencing was found to be similar for the used different concentrations as well as for concomitant application of the GABA_B_-receptor agonist (+)-5,5-dimethyl-2-morpholine acetic acid (SCH50911; 6 mM, 20 μl; n = 3) to block possible side effects of muscimol on GABA_B_ receptors [18]. In all cases cortical spiking activity is reduced by at least >95%. For further information of the adequacy of different silencing techniques see Happel et al. (2010). The same set of stimuli was presented before and after epidural application of drugs for cortical silencing.

### Current source density and residue analysis

One-dimensional current-source density (CSD) profiles were calculated from the second spatial derivative of the LFP [26,27]:

$$∼CSD\approx \frac{\delta^{2}\theta (z)}{\delta z^{2}}=\frac{\theta (z+n\Delta z)-2\theta (z)+\theta (z-n\Delta z)}{{\left(n\Delta z\right)}^{2}}$$ (1)

where *Ф* is the field potential, *z* the spatial coordinate perpendicular to the cortical laminae, *Δz* the spatial sampling interval (55–75 μm), and *n* the differentiation grid. LFP profiles were smoothed with a weighted average (Hamming window) of 5 channels (corresponding to a spatial filter kernel of 300 μm; linear extrapolation of 2 channels at boundaries). We defined the layer-dependent main sink components based on the architecture of primary input from the auditory part of the thalamus–the ventral part of the medial geniculate body (vMGB). Projections from vMGB terminate on small pyramidal neurons with local dendritic arbors and ramifying axons in cortical layers IIIb and IV, as the equivalent of spiny stellate neurons in for instance visual cortex [28–31]. These main inputs layers are henceforth generally referred to as the granular inputs layers, and layers above (I-IIIa) or beyond (V-VI) as supragranular and infragranular, respectively [32]. Corresponding sink activity was referred to as follows: early granular sink (S1), subsequent supragranular sink (S2) and infragranular sink (S3). Peak amplitudes and onset latencies of individual current sinks were determined for individual channels and then averaged. Onset latencies were determined using a linear fit around the point where each curve surpasses 3 standard deviations above/below baseline [20]. Frequencies evoking maximal responses, which we call the FMR, and shortest mean onset latencies (defined as the best frequency; BF) of the granular sink were highly correlated. Response threshold was determined as the lowest intensity eliciting a significant response at any frequency 2SD over baseline (> 5ms). Response threshold was at 20 dB SPL or higher. The frequency evoking the most prominent response (peak amplitude, onset latency) at response threshold was taken as the characteristic frequency (CF). All averaged data was pooled relative to the sound intensity at the response threshold (0–50 dB > thr). Response bandwidths were quantified as the Q10dB- and Q40dB-values separately calculated for higher frequencies (Q10/40dB_HF_) and lower frequencies (Q10/40dB_LF_) to the FMR at 10 dB and 40 dB above response threshold, respectively.

Based on single trial CSD profiles without spatial filtering we transformed the CSD by rectifying and averaging waveforms of each channel (n) comprising the laminar CSD profile (AVREC)–see Eq 2. The AVREC waveform provides a measure of the temporal pattern of the overall strength of transmembrane current flow [33,34]. The relative residue of the CSD (RelResCSD), defined as the sum of the non-rectified divided by the rectified magnitudes for each channel (Eq 3), was used to quantify the balance of the transmembrane charge transfer along the recording axis [35]:

$$AVREC=\frac{\sum_{i=1}^{n}\left|{CSD}_{i}|(t\right)}{n}$$ (2)

$$RelResCSD=\frac{\sum_{i=1}^{n}{CSD}_{i}(t)}{\sum_{i=1}^{n}\left|{CSD}_{i}|(t\right)}$$ (3)

**Fig 1:**
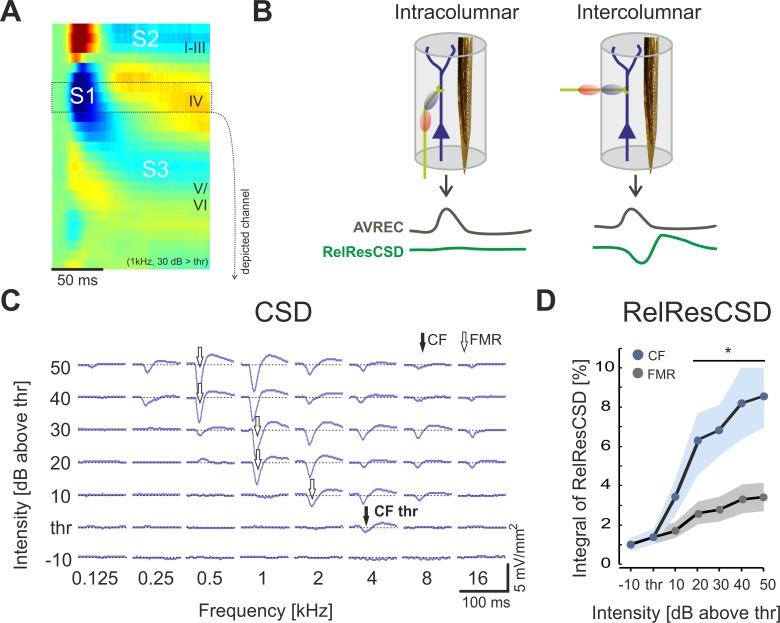
Current source density analysis of level-dependent pure-tone evoked responses in primary auditory cortex field AI. **A.** Temporal development of the dominant current sources and current sinks (S1, S2, S3) in different cortical laminae. **B**. Schematic representation of the mutually orthogonal orientation of thalamocortical (vertical) and corticocortical (horizontal) projection systems into a cortical column. Both projection systems contribute potentially different to the relative residuum of the CSD measured with a linear electrode array oriented perpendicular to the cortical surface. See also Methods and Happel et al. (2010). **C**. CSD-traces from recordings within granular layer IV from the initial current sink S1 (cortical depth ~650 μm; see inset) show level-dependent tuning changes. With increasing stimulus intensity the frequency eliciting the FMR (white arrows) was always shifted to lower frequencies (by 3 octaves in the example shown). Shifts above the CF have not been observed in any case. **D.** Potential contributions of horizontal corticocortical inputs during early input processing (first 20 ms after tone onset) revealed by RelResCSD showed a level-dependent shift of the frequency (FMR) eliciting the most balanced activation, as indicator for strongest thalamocortical input. CF-evoked responses otherwise showed increased unbalanced CSD-profiles indicative for increase in contributions of early horizontal input. Note that overall evoked activity was always highest for the FMR (see A.).

Both neuronal observables allow to dissociate the contribution of thalamocortical and intracortical projections based on their orthogonal orientation to each other. This could be followed as presynaptic terminals significantly contribute to the LFP [36,37]. Wide-spread corticocortical currents would hence be more likely distributed beyond the integration cylinder surrounding the electrode array in which extracellular currents would most contribute to the measured LFP (Fig 1B). Henceforth, only corticocortical projections should yield to deflections of the relative residues of the CSD as measured with a linear electrode array oriented perpendicular to the cortical surface [20].

We quantified the RMS values of both parameters over the first 20 ms of tone-evoked activity (onset-latency corrected) to quantify early corticocortical contributions to the evoked patterns. The temporal relationship between the averaged rectified CSD and the relative residues was quantified by comparing the onset latencies of AVREC and RelResCSD. Onset latencies were defined as the first time after stimulus onset where the waveform crossed the 3 standard deviations threshold from baseline for at least 10 ms. Baseline values were calculated based on 200 ms prior to stimulus presentation. Latencies smaller than 12 ms and longer than 60 ms were excluded from further analysis. To compare the temporal relationship of both signals, we calculated the mean (±SEM) latency difference by subtracting the onset latency of the AVREC waveform from the RelResCSD: RelResCSD_Onset_−AVREC_Onset_.

### Statistical analysis

Comparison of multiple groups was performed by multifactorial repeated-measures ANOVAs (rmANOVAs). For comparison between two groups, paired sample Student’s t tests were used. Generally, a significance level of α = 0.05 was chosen.

## Results

We have investigated the level-dependence of temporally precise convergent thalamocortical and horizontal corticocortical inputs at recording sites with defined tuning within the tonotopic map of primary auditory cortex field AI. Laminar current source density recordings were used to analyze neuronal responses to pure tones presented at sound levels from -10 dB to +50 dB above the LFP response threshold in steps of 10 dB. Thalamocortical and corticocortical inputs were dissociated by CSD residual analysis and pharmacological silencing of corticocortically relayed activity using topical application of the GABA_A_-agonist muscimol [20,21]. We found consistent changes of spectral representation with increasing sound level throughout cortical layers. Cortical layers receiving direct afferent thalamic input mainly inherited the subcortical intensity-frequency tuning, while upper layers showed a tonotopic tuning less dependent on sound level.

### Effect of sound intensity on spectral inputs provided by thalamocortical and corticocortical projections

Fig 1A shows a representative example of a pure-tone evoked current-source density (CSD) profile over time through the cortical laminae of primary auditory cortex field A1. The dominant graunular sink S1 reflects a superposition of a number of extracellular currents due to recruitment of thalamocortical and intracortical projections [20,21]. Fig 1B schematizes the effects of the geometrical arrangement of projection systems into a cortical column on the AVREC and the RelResCSD (Givre et al., 1994; Harding, 1992; Schroeder et al., 1998).

Unbalanced CSD amplitudes within the integration cylinder of a linear recording track are putatively due to presynaptic current contributions from corticocortical projections that are orthogonal to the recording axis [36,37]. Recently, we could confirm that the relative residual CSD provides a quantitative measure of the contribution from horizontal corticocortical input to the neuronal activity at a given cortical patch [20,21]. See Materials & Methods and Fig 1B for further explanation.

At each recording site (n = 8) increasing the sound intensity above threshold shifted the frequency of maximum response (FMR) from the characteristic frequency (CF) to lower frequencies (representative example in Fig 1C). We used the analysis of the relative residues of the CSD to investigate the relative contributions of thalamocortically and intracortically relayed activity to this level-dependent tuning shift. Fig 1C shows the RMS amplitude of the RelResCSD over the first 20 ms of early tone-evoked activity (onset-latency corrected) after stimulation with the CF or the FMR, respectively (note that CF and FMR are identical at -10 dB and 0 dB above threshold). Notably, stimulation with the FMR led to a much shallower rise of the residual CSD with stimulus intensity as compared to stimulation with the CF (Fig 1D). This indicates that with sound intensity CF-information is relayed gradually via corticocortical inputs. The observed shift of the FMR, however, is most likely due to a level-dependent tuning shift of thalamocortical inputs in granular layers.

### Level-dependent frequency response analysis of different synaptic input systems

**Fig 2:**
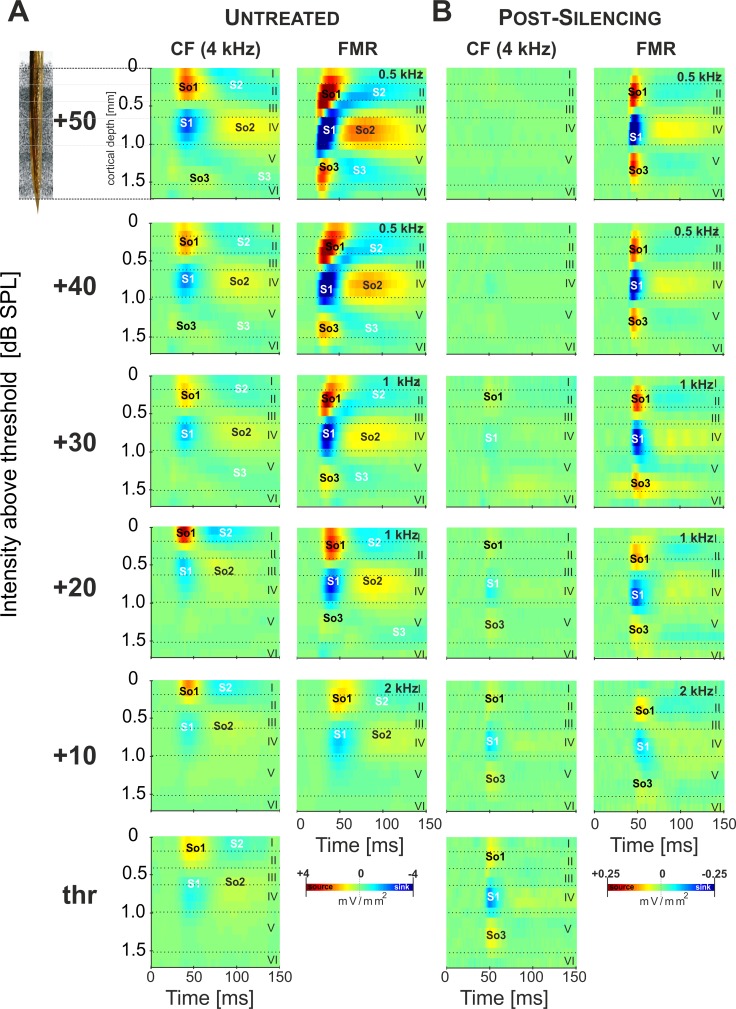
Level-dependence of pure tone-evoked laminar CSD profiles. Roman numbers indicate cortical layers. Prominent current sources (So1, So2, So3) and current sinks (S1, S2, S3) are shown in red and blue, respectively. *Inset*: custommade multichannel shaft-electrode used for recording. **A**. *Left*, pure-tone stimulation with the CF (4kHz) represented at the measurement site yielded stronger CSD amplitudes with increased sound intensity (0–50 dB above threshold), but maximal responses were systematically elicited by lower stimulation frequencies (*Right*) **B**. After cortical silencing tone-evoked activations in AI were observed mainly in thalamocortical input layer IV (>30 min after application), and CSD maxima and minima were <10% of the predrug values. Muscimol did not affect response threshold, but reduced the bandwidth of evoked responses. After cortical silencing, CF responses in the shown example displayed a non-monotonic decrease (*left*), while the FMR shifted to lower frequencies with increase in sound level (*right*). This indicates that the described BF shift in granular layers is already inherited from afferent inputs. See also Fig 3B.

**Fig 3:**
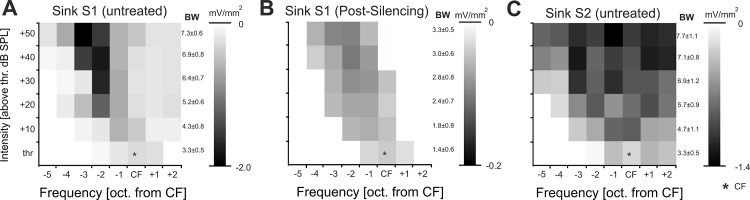
Frequency response areas (FRA) of early and late activations and quantification of FMR-shift. **A.-C.** Mean peak amplitudes of early (S1) and late (S2) current sink activity as a function of stimulation frequency and intensity referenced to the CF (black asterisk). **A.** FRA of early current sink activity in thalamocortical input layers showed increase of response bandwidth (BW) and decrease of FMR with increasing intensity. **B.** Cortical silencing reduced response bandwidth and shifts of the FMR, but did not affect response threshold. **C.** FRA of later activity in supragranular layers (S2) before cortical silencing showed increasing response bandwidth without systematic FMR-shift in dependence of the stimulus intensity.

**Fig 4:**
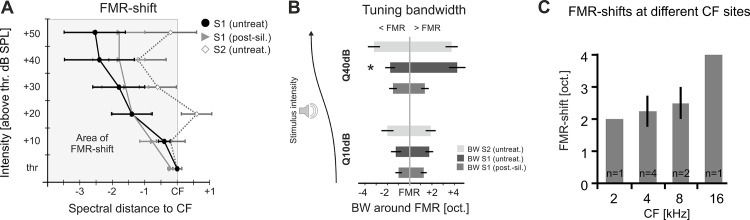
Comparison of level-dependent frequency shifts (± SEM) evoking maximal responses across animals and recordings sites. **A.** Quantitative comparison of level-dependent frequency shifts (± SEM) evoking maximal responses across animals of the granular sink S1 before (black) and after cortical silencing (grey) and the supragranular sink S2 (grey open). For statistical information see text. **B.** Quantification of tuning bandwidths of the low and high frequency region around the FMR showed a significant difference exclusively for higher sound levels (Q40dB) in early granular sink activity in untreated cortex (*p<0.05). **C.** Distribution of recording positions to corresponding CF-derived tonotopic sites in AI and consecutive FMR-shifts at 40 dB above threshold showed no significant difference between mid- and high-frequency regions. Grey bars show averaged FMR-shifts at individual CF-sites. No significant differences for the FMR-shift were found at maximal stimulation amplitudes between sites of different CF (t-test; p>0.05).

To critically test this hypothesis we isolated potential contributions of any thalamocortical input and the intracortical circuitry to level-dependent tuning shifts. Therefore, we investigated the level-dependent activation of auditory cortex with and without pharmacological blocking of corticocortically relayed activity using topical application of muscimol [20]. In untreated cortex, pure-tone evoked CSD profiles showed relatively constant amplitudes of the dominant granular sink S1 with increasing sound intensity after stimulation with the CF (4 kHz in the example shown; Fig 2A; left column and Fig 3A). With increasing intensity, maximal responses were evoked by pure-tone stimulation with frequencies below the CF (Fig 2A; right column and Fig 3A). After silencing of intracortical circuits, the tone-evoked CSD exclusively reflected the activity of any thalamocortical input. Isolated thalamocortical synaptic activity was found to largely overlap with the granular layer sink S1 and still showed a considerable FMR-shift with increasing stimulus intensity (Fig 2B and see also Fig 4A). Synaptic activity in upper layers vanished completely indicating no contribution of extracortical inputs [20].

For quantification we characterized this effect by calculating grand mean frequency-response areas (FRAs) across all animals. FRAs were compared for early granular current sink S1 amplitudes before and after cortical silencing and pre-muscimol supragranular S2 sink amplitudes (Fig 3). FRAs of S1 in untreated AI showed an increase of response bandwidth (BW) with increasing intensity mainly due to expansion of low-frequency input (Fig 3A). After intracortical silencing, evoked amplitudes of the sink S1 were significantly reduced (for FMR stimulation paired *t*-test; p<0.001; cf. [18,20]). The FRA of the S1 after silencing showed decreased response bandwidths at all intensities (*t*-test; p<0.05) but no change of response threshold or CF (paired *t*-test; p>0.05; Fig 3B). Nevertheless, the FMR also showed a level-dependent shift towards lower frequencies. Comparing the FRA of S1 and the supragranular S2 activity before silencing showed increased response bandwidths (BW) in upper layers due to increased activity across the complete range of stimulation frequencies without a systematic level-dependent FMR-shift (Fig 3C).

Quantitative analysis of the group data on level-dependent shifts (± SEM) of the FMR (Fig 4A) showed the strongest shift for the granular sink S1 before silencing (black; 2.8±1.25 octaves below CF at 50dB above threshold). Cortical silencing reduced the shift mainly for higher stimulation intensities (grey; 1.80±0.80 oct below CF at 50dB above threshold). In both untreated and silenced cortex the FMR was significantly shifted compared to the CF for sound intensities ≥20 dB above threshold (rmANOVA; significant main effect of factors “FMR shift” (F_5,25_ = 31.2; p<0.001) and “silencing” (F_1,5_ = 49.7; p<0.001); Fig 4A). FRAs of the later current sink S2 did not show a systematic shift of the FMR (rmANOVA; no main effect of factor “FMR shift” (F_1,5_ = 5; p = 0.177); Fig 4A; grey open).

We further analyzed tuning bandwidths of the 3 main sink components at low stimulation intensities (Q10dB) and higher intensities (Q40dB) relative to the FMR (Fig 4B). Bandwidth of the granular sink S1 was symmetric around the FMR at Q10dB in untreated cortex. At higher intensities bandwidth was asymmetric with a broader spectral extent towards the higher frequency side (Q40dB_HF_ > Q40dB_LF_; p<0.05). After cortical silencing this bandwidth asymmetry in granular layer activity had vanished (Q10/40dB_LF_ vs. Q10/40dB_HF_; paired *t-test*; p>0.05). Bandwidths of supragranular activity showed also a symmetric tuning around the FMR at both low and high stimulation intensities (Q10/40dB_LF_ vs. Q10/40dB_HF_; paired *t-test*; p>0.05).

To exclude any bias in these results due to specific tonotopic locations within AI, recording positions were distributed across a broad range of different CFs represented in AI. Fig 4C shows the distribution of CFs determined at the recording sites at response threshold. CF sites at mid- and high-frequency regions varied between 2 and 16 kHz and showed no significant difference in FMR-shift (rmANOVA; no main effect of factor “FMR shift” (F_1,3_ = 9; p = 0.245); Fig 4A).

### Level-dependent organization of temporally early convergent inputs

**Fig 5:**
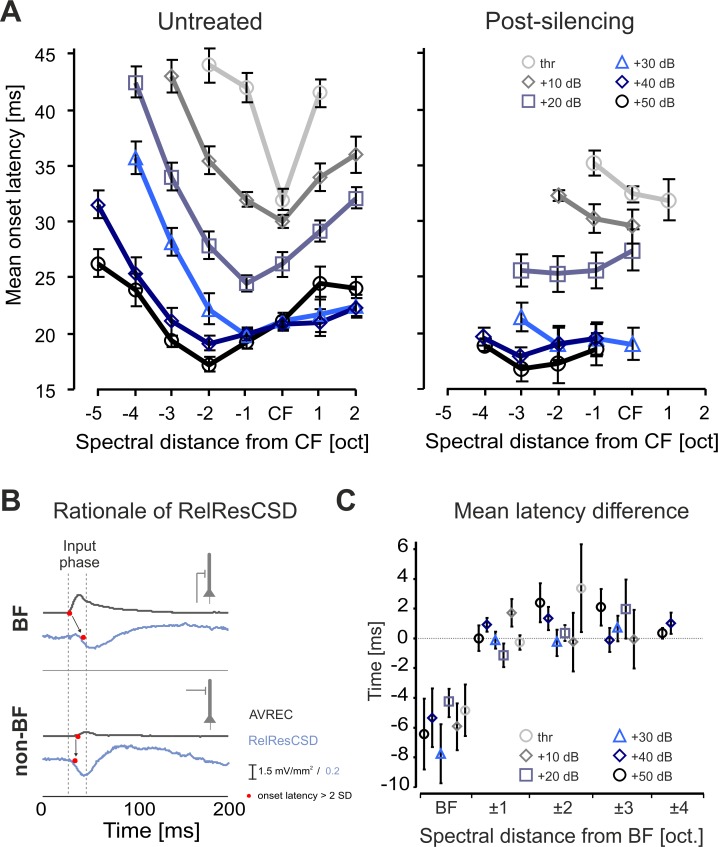
Temporal precision of thalamocortical and corticocortical synaptic inputs with varying stimulus intensity **A.** *Left*, Mean (±SEM) granular sink onset latency in untreated AI showed similar shift of the frequency evoking the earliest onset (2.1±0.85 oct. below CF), as compared to the FMR. Frequencies evoking the shortest onset or the maximal peak response were identical in most cases and differed maximally in one octave from the FMR (data not shown). *Right*, After silencing the corticocortical contribution of tone-evoked activity in AI the V-shaped onset-latency tuning was flattened to latencies comparable to CF stimulation in untreated cortex and reduced in bandwidth to CF- and near-CF-frequencies (cf. Happel et al., 2010). **B.** Schematic illustration of the mutually underlying cortical circuitry allowing to detect contributions to the evoked responses from the horizontal corticocortical circuitry in time (Happel et al., 2010). **C.** Before cortical silencing, negative mean (±SEM) latency difference of *RelResCSD onset latency–AVREC onset latencies* indicating temporal lead of the thalamocortical input over lateral corticocortical input was only found for BF stimulation. When stimulating with pure tones in spectral distance to the BF those differences were vanished, indicative for a temporally highly precise integration of substantial corticocortical contributions.

We previously reported a temporally precise convergence of thalamocortical and early lateral corticocortical inputs underlying the initial activation of a cortical site [20]. Only when stimulating with the BF, thalamocortical input was leading the lateral input in time. Thus, the onset latency of the granular sink S1 provides a robust physiological indicator for the frequency evoking the strongest thalamocortical input. In the present study, averaged mean onset latencies (±SEM) showed a shift of the frequency evoking the earliest onset towards frequencies below the CF with increasing intensity (Fig 5A *left*; 2.1±0.85 oct. below CF at 50 dB > thr). Best frequencies evoking the shortest onset latency differed maximally by 1 octave from the FMR (data not shown). In agreement with former reports we found no change in onset latency for the BF by cortical silencing. However, muscimol mainly decreased onset latencies for frequencies near the BF resulting in a flattened latency tuning curve irrespective of the sound intensity (Fig 5A, *right*).

In order to quantify the functional contribution of specifically lateral corticocortical input to the described effect, we compared the time courses of the RelResCSD (blue) and of the AVREC (black) (Fig 5B). For BF stimulation, RelResCSD onset latency was found to be longer than that of the AVREC, indicating temporal lead of the thalamocortical input over lateral corticocortical input. However, at other stimulation frequencies no such latency difference was found. Most notably, this coincidence of thalamocortical and lateral corticocortical input for frequencies already 1 octave away from the BF, was independent of the sound level and also accounted for the CF (Fig 5C).

## Discussion

The functional anatomy of thalamocortical projections in primary auditory cortex field AI provides spectrally broad input to a given cortical site within the tonotopic map of AI [18,19,38]. Hence, a given cortical patch is expected to respond to pure tones of frequencies spanning several octaves depending on sound level [39,40]. Generally, the CF at a given cortical patch is used to define the tonotopic region of recording.

However, the presented data demonstrates that the CF gives only limited information about the tonotopic representation of sound frequency at a specific recording location in AI above response threshold. The present study described a level-dependent integration of thalamocortical and early lateral corticocortical inputs within the granular layers of AI: With increasing stimulus intensity the synaptic population at a particular tonotopic patch carrying information about the CF shifts from thalamocortical input to mainly corticocortical inputs, while thalamocortical inputs convey spectral information of lower frequencies (Fig 1B). The observed shift of the FMR from CF to lower stimulation frequencies is most likely due to thalamocortically relayed rather than corticocortically relayed inputs. This challenges the suitability of the CF for characterizing the functional topography of the thalamocortical projection at a given cortical site with respect to changing sound levels (see Fig 2B).

### Sound level changes spectral representations throughout the auditory pathway

Peripheral auditory transduction in vertebrates is based on the mechanics and nonlinear filter characteristics of the basilar membrane and active amplification via the hair cells in the cochlea [41]. Modelling approaches predict that the peripheral transduction processes in vertebrates yield a decrease of the frequency evoking the highest response (FMR) with increasing sound levels [42]. This has been discussed to reflect a protection mechanism for CF regions against overstimulation due to resonant absorption [43] or a mechanism for optimal tuning to conspecific sounds [44]. Physiological studies of peripheral auditory nerve fiber (ANF) responses in vertebrates have indeed found that best frequencies of individual fibers shifted mainly to lower frequencies with increasing stimulus intensities [3,45,46]. In agreement, psychophysical tuning curves in humans revealed a general decrease of the best frequency with increasing sound levels across the entire hearing range [47].

Yet, recordings from single neurons along the central auditory pathway often reveal a robust best frequency across sound levels. Nevertheless, a smaller percentage of single neurons that show a decreasing BF with increasing sound levels were consistently reported in the dorsal and ventral cochlear nucleus (DCN/VCN; [4,48]), the inferior colliculus (IC; [49–53]), and the auditory thalamus (MGB; [54,55]). Comparison of the peripheral transfer-function of the basilar membrane, ANF firing patterns and single neuron response types found in the central auditory system suggests central compensation mechanisms yielding level-tolerant or wide-dynamic range tuning of individual neurons. In cochlear nucleus it has been demonstrated that neurons discharging precisely at stimulus onset are more likely to show decreasing BFs with increasing sound level (type I), while units with sustained activity showed rather little changes of the shape of the CF-rate level function [4,56]. The authors have discussed that first impulse responses more likely reflect BF shifts inherited from the peripheral transduction process, while local processing within the CN compensates for the effect. Similarly, local circuits in the IC substitute afferent inherited inputs with increasing sound level in order to shape the central sound-intensity code [57].

### Layer specificity of level dependent spectral integration in auditory cortex

Our data suggests that the earliest response components in middle thalamocortical-recipient layers of auditory cortex originating from the ventral MGB also reflects the intensity-tuned frequency representation. Pharmacologically isolated thalamocortical inputs in granular layers were consistently shifted towards lower frequencies with increasing sound intensity (Fig 3B). Response bandwidth and response strength, however, only showed moderate increases (Fig 3B). Hence, the full range of frequency information carried by thalamocortical synapses in granular layers of auditory cortex is fully mapped only when stimulating with varying sound level. This is in accordance with whole-cell recordings in thalamocortical recipient cells in auditory cortex, which have shown that non-monotonic firing behavior can be inherited from thalamocortical afferents [5,58]. Before cortical silencing, granular sink activity was significantly stronger and more broadly tuned (Fig 3A). Also, at intensities 30dB above threshold this FMR shift was even slightly higher before cortical silencing (Fig 4A). This might reflect the potential amplification of afferent inputs by local recurrent excitation [18].

The described level-dependence of early granular synaptic inputs could not be demonstrated for later supragranular activity (sink S2) in our data set (Fig 4A). Subsequent synaptic activation in supragranular layers has been mainly related to corticocortical local and long-range synaptic inputs [20,59]. The fact that we did not observe any synaptic input in layers I-IIIa after cortical silencing largely rules out a significant contribution from non-lemniscal thalamic inputs. Using the residual CSD analysis we could further identify a high-threshold component of lateral corticocortical input that converges with the input inherited from the subcortical auditory pathway. The discrepancy of granular and supragranular level-dependent tuning profiles suggests a corticocortical integration of spectral and intensity information in upper layers at a given cortical pitch. Thereby, intercolumnar synaptic integration across frequency channels potentially reflects the cortical correlate of the circuit-based compensation of the peripheral sound-intensity transduction [57]. Our model suggests an asymmetric flow of information by increase in sound level preferentially higher-frequency tonotopic regions towards lower-frequency regions and is hence in agreement with recent functional anatomical data using laser scanning photostimulation [60]. Intracortically routed activity in supragranular layers is thereby gain-modulated by sound intensity, but intensity-constant with respect to tuning. This might explain the occurrence of neurons that are intensity-tuned or have a wide-dynamic range as a result of distinct local or wide-range integrating synaptic microcircuits. Thereby, the presented synaptic population data of individual input systems is in accordance with recent anatomical findings [61]. Further, data from 2-photon imaging of single-cell spectral responses showed that is depends on laminar depth, afferent or intracortical inputs and hence the cortical processing hierarchy [16,17,62,63]. Corticocortical integration in neurons mainly receiving broad corticocortical inputs might hence contribute to the perceptual robustness across sound levels based on population coding [9,10,15].

### Temporally precise integration of thalamocortical and corticocortical spectral inputs

Comparison of the onset latencies between the AVREC and the RelResCSD yields a measure of lateral cortico-cortical input at a given cortical site (see Materials & Methods and Fig 5B). In agreement with previous reports, we found that both AVREC and RelResCSD possess the same onset latencies when the stimulation frequency is not the BF. For BF stimulation, however, onset latencies of the AVREC are about 4–8 ms shorter (Fig 5C). This indicates that CF-responses at higher sound intensities in untreated cortex (Fig 2A) dominantly reflect early corticocortically routed synaptic inputs, as determined by the instantaneous unbalanced CSD residuum [20]. Our results thereby confirm that the anatomically overlapping thalamocortical and intracortical synaptic circuits converge in order to generate the sound-intensity tuning in granular input layers [64,65].

Interestingly, cortical silencing abolished the V-shaped onset latency tuning of the granular sink suggesting that lateral inputs might precisely modulate the feedforward activation cascade of neighboring cortical sites [66]. Response latency might be controlled via inhibitory mechanisms, as for instance thalamocortical feedforward inhibition [67,68] or lateral sharpening of balanced inhibition [69,70]. For a detailed discussion of lateral inputs in thalamocortical-recipient layers to control and integrate excitatory afferent input to non-optimal stimulation see [20,58].

Importantly, we found that CF-responses at higher sound intensities in untreated cortex were dominantly reflected by early corticocortically routed synaptic inputs, as determined by the instantaneous unbalanced CSD residuum (Fig 5C). We therefore propose that increase of sound level presumably led to enhanced activation of cortical sites with higher CF promoting afferent corticocortical input from higher to lower frequency sites. This interpretation is further supported by the observed asymmetric bandwidth tuning around the FMR of early intracortical responses (S1 untreated; Fig 4B) when stimulating with 40 dB above response threshold: high-intensity tuning showed a broader extent towards the higher CF-frequency site as compared to off-BF-frequencies in the low-frequency range. In contrast, isolated thalamocortical input did not show such asymmetric bandwidth around the FMR (Fig 4B). This suggests a cortical origin of the asymmetric high-intensity tuning bandwidth towards the CF due to lateral corticocortical processing (Fig 5C) in accordance with recent data [60]. Thus, intercolumnar circuits may allow intensity integration processes across the tonotopic gradient by lateral propagation of activity between low- and high-frequency regions [60,71]. Consistently, we found an increase of the RMS of the RelResCSD with sound level (see Fig 1B). Early lateral inputs in granular layers might therefore serve as a synaptic connection between cortical circuits mediating threshold-near CF-responses and maximal responses with higher intensities. The temporally precise convergence of thalamocortical and corticocortical input systems allows the integration of spectral inputs readjusted to a given sound level. This could therefore provide a circuit basis for spectral integration of the different stimulus components contributing to a complex sound.

### Relevance of spatial location within the tonotopic gradient of AI

In this study, we sampled data from different locations across the tonotopic gradient of AI from middle- (1–4) to high-frequency (8–16) CF-sites (Fig 4B). As judged by near-threshold LFP-responses we did not find CFs below 1 kHz in the here described experiments, nor in other experiments with similar stimulation parameters. Thereby, CF sites investigated in this study fell into the optimal hearing range between 2 to 4 kHz of the Mongolian gerbil [72,73]. In our data set, enhanced responses to frequencies below the CF with increasing sound intensity were independent of the tonotopic location. This is in agreement with former reports throughout the auditory pathway from auditory nerve fibers [3] to auditory cortex [19]. Hence, such shifts could not be explained as a mere result of the u-shaped hearing threshold profile [47].

### Sound level dependence changes by hierarchical columnar processing

Considerable changes of frequency tuning curves of unit data from AI at higher sound levels including shifts in best frequency have been described earlier and are commonly interpreted as a result of broader spectral tuning [2]. In this study, we identified explicit synaptic integration processes within different layers of a cortical column in dependence of the spectral and level information of acoustic stimuli. Importantly, we found that on a population level thalamocortical inputs are co-tuned to frequency and intensity: for a given frequency, responses are only evoked over a range of intensities. In supragranular layers we found no level-dependent changes of synaptic population tuning, but more constantly albeit broadly tuned responses with increasing sound level (Figs 3C and 4A). This finding might potentially explain the occurrence of level-tuned neurons and level-robust neurons as a result of hierarchical cortical processing of spectral and level information [11].

Interestingly, Pienkowski and Eggermont (2011) have reported a level-invariant representation of the spectral energy of acoustic stimuli based on MUA and LFP signals only for responses to complex sounds, but not for single tone pips, as used in this study [74]. Due to the spectral energy distribution of complex tones it has been suggested that competitive interactions between cortical columns account for the spectral integration [14,75]. Such processes would be strongly reflected in supragranular sink activity [20,21,59,76]. Early bottom-up synaptic inputs in AI might be driven most effectively by single tone pips, while later corticocortical processing in upper layers would be more effectively activated by complex tones. Therefore, we hypothesize that the laminar position and the respective role within the columnar processing of a given cortical cell in AI is relevant for the individual sound level response properties. Such hierarchical processing might also underlie the findings in human imaging studies that described an ampliotopic representation of spectral information for early dipole currents of auditory evoked potentials in auditory cortex with varying stimulus intensity [77] but level-invariant representation of spectral content in longer temporal evoked responses [78].

Notably, main cortical efferent outputs to numerous subcortical and other cortical targets are provided by infragranular LV pyramidal neurons. Apical dendrites of LV neurons extend through all layers and, hence, do receive inputs in supragranular layers. Infragranular pyramidal neurons might thereby integrate spectral information by level-tuned early synaptic inputs and broadly tuned level-invariant inputs via distinct dendritic target regions [79]. The described hierarchical columnar integration of spectral and intensity information might allow the proper adjustment of auditory perceptual representations in situations of variable stimulus amplitudes [80,81]. Such functional cortical circuitry within auditory cortex might be fundamental for a constant representation of ecologically relevant auditory objects over a large range of intensities within noisy acoustic environments [10,11,82].
